# Association of Combined Patterns of Tobacco and Cannabis Use in Adolescence With Psychotic Experiences

**DOI:** 10.1001/jamapsychiatry.2017.4271

**Published:** 2018-01-17

**Authors:** Hannah J. Jones, Suzanne H. Gage, Jon Heron, Matthew Hickman, Glyn Lewis, Marcus R. Munafò, Stanley Zammit

**Affiliations:** 1 Centre for Academic Mental Health, Population Health Sciences, Bristol Medical School, University of Bristol, Bristol, United Kingdom; 2 Medical Research Centre, Integrative Epidemiology Unit, University of Bristol, Bristol, United Kingdom; 3 Department of Psychological Sciences, University of Liverpool, Liverpool, United Kingdom; 4 Division of Psychiatry, University College London, London, United Kingdom; 5 UK Centre for Tobacco and Alcohol Studies, School of Experimental Psychology, University of Bristol, Bristol, United Kingdom; 6 MRC Centre for Neuropsychiatric Genetics and Genomics, Division of Psychological Medicine and Clinical Neurosciences, Cardiff University School of Medicine, Cardiff, United Kingdom

## Abstract

**Importance:**

There is concern about potentially causal effects of tobacco use on psychosis, but epidemiological studies have been less robust in attempts to minimize effects of confounding than studies of cannabis use have been.

**Objectives:**

To examine the association of patterns of cigarette and cannabis use with preceding and subsequent psychotic experiences, and to compare effects of confounding across these patterns.

**Design, Setting, and Participants:**

This cohort study used data from the Avon Longitudinal Study of Parents and Children, which initially consisted of 14 062 children. Data were collected periodically from September 6, 1990, with collection ongoing, and analyzed from August 8, 2016, through June 14, 2017. Cigarette and cannabis use data were summarized using longitudinal latent class analysis to identify longitudinal classes of substance use. Associations between classes and psychotic experiences at age 18 years were assessed.

**Exposures:**

Depending on the analysis model, exposures were longitudinal classes of substance use or psychotic experiences at age 12 years.

**Main Outcomes and Measures:**

Logistic regression was used to examine the associations between substance use longitudinal classes and subsequent onset of psychotic experiences.

**Results:**

Longitudinal classes were derived using 5300 participants (56.1% female) who had at least 3 measures of cigarette and cannabis use from ages 14 to 19 years. Prior to adjusting for a range of potential confounders, there was strong evdience that early-onset cigarette-only use (4.3%), early-onset cannabis use (3.2%), and late-onset cannabis use (11.9%) (but not later-onset cigarette-only use [14.8%]) latent classes were associated with increased psychotic experiences compared with nonusers (65.9%) (omnibus *P* < .001). After adjusting for confounders, the association for early-onset cigarette-only use attenuated substantially (unadjusted odds ratio [OR], 3.03; 95% CI, 1.13-8.14; adjusted OR, 1.78; 95% CI, 0.54-5.88), whereas those for early-onset cannabis use (adjusted OR, 3.70; 95% CI, 1.66-8.25) and late-onset cannabis use (adjusted OR, 2.97; 95% CI, 1.63-5.40) remained consistent.

**Conclusions and Relevance:**

In this study, our findings indicate that while individuals who use cannabis or cigarettes during adolescence have an increased risk of subsequent psychotic experiences, epidemiological evidence is substantively more robust for cannabis use than it is for tobacco use.

## Key Points

**Question:**

Are patterns of adolescent cigarette and cannabis use differentially associated with subsequent onset of psychotic experiences?

**Findings:**

In this longitudinal cohort study of 3328 adolescents, there is evidence that both cannabis and cigarette use are associated with subsequent psychotic experiences prior to adjusting for confounders. However, after adjusting, the associations for cigarette-only use attenuated substantially, whereas those for cannabis use remained consistent.

**Meaning:**

While individuals who use either cannabis or cigarettes during adolescence appear to be at increased risk of psychotic experiences, the association of psychotic experiences is greater with cannabis than with tobacco smoking.

## Teaser

This cohort study investigates whether patterns of tobacco and cannabis use in adolescents are associated with psychotic experiences.

## Introduction

Cannabis and tobacco are frequently used together, identifying their individual associations on mental health is difficult but important, as this can advance understanding of causal mechanisms and help target preventive interventions.

Individuals who use cannabis regularly have a 2- to 3-fold increased risk of a psychotic outcome.^1^ Tobacco use is also associated with an increased incidence of psychotic disorders^2,3,4,5^ in cohort studies, and (less consistently) with subclinical psychotic symptoms,^6,7,8^ with hypothesized casual mechanisms including nicotine increasing dopamine release and inducing D_2_-receptor supersensitivity.^5,9^

However, while a recent systematic review reported a meta-analysis estimate for daily smoking and psychosis that was similar to that for regular cannabis use, the estimate was based on results unadjusted for confounders,^5^ unlike that for cannabis.^1^ While concern about confounding leading to overestimation of association on psychosis also exists for cannabis,^1^ support for causal effects of cannabis also comes from experimental studies showing an increase in psychotic experiences following exposure to intravenous Δ9-tetrahydrocannabinol (THC).^10^ In contrast, experimental studies of nicotine administration do not support the acute onset of psychotic experiences.^11^

The strongest evidence of a causal effect of tobacco on psychosis is that a genetic locus associated with heaviness of smoking (within the nicotinic receptor *CHRNA5-A3-B4* gene cluster) is 1 of the loci most strongly associated with schizophrenia.^12^ However, this is also theoretically consistent with either confounding by shared genetic effects (biological pleiotropy) or, perhaps less plausibly, reverse causality (ie, biological risk of schizophrenia causing smoking behavior).

Associations between genetic risk for psychosis and both cannabis use and heaviness of cigarette use are also consistent with causal effects, reverse-causal effects, and pleiotropy explanations.^13,14,15^

As most people who use cannabis also smoke cigarettes, teasing out potentially causal effects of cannabis from those of tobacco is difficult, particularly as individuals usually mix their cannabis with tobacco, even when classing themselves as nonsmokers.^16^ Measurement error can lead to incorrect estimates of causal effects (see Gage et al^16^ and Munafò et al^17^ for examples of the impact of measurement error on confounding and main effects) and is particularly likely when using single–time point assessments of exposure status. Thus, other methods for teasing out causal effects of cannabis as distinct from tobacco are required.

One approach that can help inform causal inference is to use behavioral patterns of cannabis and cigarette use over time to identify classes of individuals with different substance use profiles across a developmental period rather than relying on patterns of cannabis and cigarette use at a single point in time.^18^ Such methods capture additional information that may enable continual users of cannabis and cigarettes to be distinguished from those who may have experimented briefly.

In this study, we used longitudinal latent class analysis (LLCA) to identify subgroups of individuals based on similar patterns of cigarette and cannabis use behavior over time to examine the association of different classes with subsequent onset of psychotic experiences, compare patterns of confounding across these classes, and examine the association of childhood psychotic experiences with adolescent patterns of cigarette and cannabis use.

## Methods

### Participants

The sample comprised individuals within the Avon Longitudinal Study of Parents and Children birth cohort. The initial cohort consisted of 14 062 children born to women residing in the former Avon Health Authority area with expected delivery dates from April 1, 1991, to December 31, 1992.^19,20,21^ All participants provided written informed consent, and ethical approval was obtained from the Avon Longitudinal Study of Parents and Children Ethics and Law Committee and the local research ethics committees.

### Measures

#### Cigarette and Cannabis Use

Measures of cigarette and cannabis use were collected at 6 time points between ages 14 and 19 years (eAppendix in the Supplement). As very few individuals used cannabis without tobacco^16^ (eTable 1 in the Supplement), data at each time point were summarized as individuals who did not report cigarettes or cannabis use, individuals who reported cigarette use only, and individuals who reported cannabis use (with or without cigarettes).

#### Psychotic Experiences

The semi-structured psychosis-like symptom interview (PLIKSi)^22,23^ was used to assess psychotic experiences at ages 12 and 18 years. The PLIKSi allows rating of 12 psychotic experiences including hallucinations, delusions, and thought interference.

The primary psychotic experience measures at ages 12 and 18 years were binary variables relating to whether an individual had at least 1 definite psychotic experience compared with suspected or no psychotic experiences. As sensitivity analyses, we also repeated analyses using narrower (definite psychotic experiences vs none; psychotic disorder vs none) and broader (definite or suspected psychotic experiences vs none) cutoffs for defining the outcome (eAppendix in the Supplement).

#### Potential Confounders

Potential confounders examined included sex, family history of schizophrenia or depression, family history of drug use, maternal and/or paternal smoking during pregnancy, maternal education, highest parental social class, IQ (age 8 years), childhood trauma or experiencing bullying (ages 7-9 years), emotional and behavioral problems (Strengths and Difficulties Questionnaire score, age 9 years), and alcohol use (age 12 years) (eAppendix in the Supplement).

### Statistical Analysis

#### Longitudinal Latent Class Analysis

Longitudinal latent class analysis was used to derive distinct behavior patterns in the repeated-measures data relating to cigarette and/or cannabis use as previously described.^24,25^ The aim of LLCA is to identify the number of latent classes that adequately explain the relationship between the observed variables. Individuals were included in the analysis if they had data present for 3 or more time points. Starting with 1 class, additional classes were added and the model fit was assessed until the optimal number of classes was achieved. Model fit was assessed using the following parameters: proportion of individuals in each class, sample size–adjusted Bayesian information criterion, and Lo-Mendell-Rubin likelihood ratio test. Longitudinal latent class analysis was performed using MPlus version 7.31 software.^26^

#### Association Analyses

##### Psychotic Experiences as Exposure

Multinomial regression was used to assess whether psychotic experiences at age 12 years were associated with subsequent latent class membership, before and after adjustment for potential confounders, using a manual implementation of the bias-adjusted 3-step method (eAppendix in the Supplement).^27^ Analyses were also conducted on a restricted sample omitting 455 participants who used cannabis or cigarettes at age 12 years.

##### Psychotic Experiences as Outcome

Logistic regression was used to assess whether latent class membership was associated with subsequent psychotic experiences at age 18 years, before and after adjustment for potential confounders. For these analyses, derivation of classes was restricted to data from the first to fourth time point (approximate ages 14-17 years). Otherwise, the method used to derive classes was as previously described. Restricting data to 4 time points had minimal impact on latent class structure and proportions (eFigure and eTable 2 in the Supplement). Analyses were also conducted on a restricted sample omitting 149 participants with definite psychotic experiences at age 12 years.

Adjusting for family history of schizophrenia or depression, family history of drug use, paternal smoking during pregnancy, social class, IQ, experiencing bullying, childhood trauma, and alcohol use had almost no association with results for either model previously described (eTable 3 in the Supplement) but reduced the analysis sample size substantially. We therefore only adjusted for sex, maternal education, maternal smoking during pregnancy, and child Strengths and Difficulties Questionnaire score in our final adjusted model.

#### Missing Data

Percentage of missing data increased with time (eTable 4 in the Supplement). Participants in the analysis sample were more likely to be female and to come from more advantaged backgrounds (Table 1 and eTable 5 in the Supplement).

**Table 1.** Sample Demographics for Participants Who Completed Questions Related to Cigarette and Cannabis Use per Time Point^a^

Time Point	Data Source	Respondents/Time Point, No.	Female, No. (%)	Age, y	
				Mean	Median (Range)
1	Interview	4654	2530 (54.4)	13.8	13.8 (12.5-15.2)
2	Postal questionnaire	4537	2608 (57.5)	14.2	14.1 (14-16.2)
3	Interview	4421	2421 (54.8)	15.4	15.3 (14.3-17.5)
4	Postal questionnaire	4169	2478 (59.4)	16.7	16.6 (16.4-18.1)
5	Interview	3541	2002 (56.5)	17.7	17.7 (16.3-19.6)
6	Postal questionnaire	2927	1878 (64.2)	18.6	18.7 (17.8-20)

^a^ Approximate age used to plot the data was 14 years for time point 1, 15 years for time point 2, 16 years for time point 3, 17 years for time point 4, 18 years for time point 5, and 19 years for time point 6.

## Results

Data were available for 5300 participants (56.1% female). Based on model fit statistics (eTable 6 in the Supplement), there was good agreement that a 5-class solution adequately described the heterogeneity within the data.

The 5-class model comprised individuals with a higher probability of early-onset cigarette-only use (4.3%), early-onset cannabis use (3.2%), late-onset cigarette-only use (14.8%), late-onset cannabis use (11.9%), and individuals with a very low probability of cigarette or cannabis use (65.9%; referred to as nonusers) (Figure).

**Figure.**
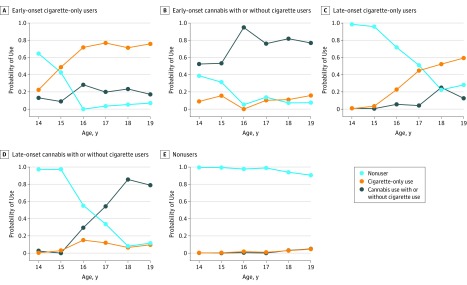
Five-Class Model of Cigarette and Cannabis Use Patterns From a Sample of 5300 Participants The probability axis represents the probability of a class member being a nonuser, a cigarette-only user, or a cannabis with or without cigarette user at each time point.

### Patterns of Cigarette and/or Cannabis Use at Ages 14 to 17 Years and Psychotic Experiences at Age 18 Years

Individuals within the early-onset cigarette-only class, but not the late-onset cigarette-only class, had greater odds of psychotic experiences at age 18 years when compared with nonusers (odds ratio [OR], 3.03; 95% CI, 1.13-8.14; and OR, 0.84; 95% CI, 0.31-2.31, respectively) ( Table 2).

**Table 2.** Associations Between Cigarette and/or Cannabis Use and Psychotic Experiences at Age 18 Years

Variable	Definite Psychotic Experiences (n = 3328)			
	Unadjusted		Adjusted	
	OR (95% CI)^a^	*P* Value^b^	OR (95% CI)^a^^,^^c^	*P* Value^b^
Early-onset		<.001		<.001
Cigarette-only	3.03 (1.13-8.14)		1.78 (0.54-5.88)	
Cannabis	3.79 (1.73-8.31)		3.70 (1.66-8.25)	
Late-onset				
Cigarette-only	0.84 (0.31-2.31)		0.73 (0.27-1.98)	
Cannabis	3.05 (1.69-5.53)		2.97 (1.63-5.40)	

Abbreviation: OR, odds ratio.

^a^ Compared with nonusers class.

^b^ The omnibus *P* value for associations between cigarette and/or cannabis use classes and psychotic experiences at age 18 years.

^c^ Adjusted for sex, maternal education, emotional and behavioral problems (Strengths and Difficulties Questionnaire score at age 9 years), and maternal cigarette smoking during pregnancy.

There was strong evidence that participants within the early-onset and late-onset cannabis use classes also had increased odds of psychotic experiences (early-onset cannabis use: OR, 3.79; 95% CI, 1.73-8.31; late-onset cannabis use: OR,3.05; 95% CI, 1.69-5.53).

When adjusting for confounding, the evidence of association between early-onset cigarette-only use and psychotic experiences was attenuated by approximately 60% (adjusted OR, 1.78; 95% CI, 0.54-5.88) (Table 3). In contrast, adjusting for confounding had minimal impact on the associations for early-onset cannabis use (adjusted OR, 3.70; 95% CI, 1.66-8.25) or late-onset cannabis use (adjusted OR, 2.97; 95% CI, 1.63-5.40).

**Table 3.** Associations Between Psychotic Experiences at Age 12 Years and Subsequent Cigarette and/or Cannabis Use

Definite PE	OR (95% CI)^a^				*P* Value^b^
	Early-Onset		Late-Onset		
	Cigarette	Cannabis	Cigarette	Cannabis	
Unadjusted (n = 4101)	1.17 (0.41-3.33)	0.97 (0.31-3.00)	1.76 (1.01-3.10)	1.66 (0.94-2.91)	.14
Adjusted (n = 4101)^c^	0.86 (0.27-2.81)	0.93 (0.28-3.06)	1.60 (0.91-2.82)	1.65 (0.90-3.05)	.25

Abbreviations: OR, odds ratio; PE, psychotic experiences.

^a^ Compared with nonusers class.

^b^ The omnibus *P* value for association between psychotic experiences at age 12 years and cigarette and/or cannabis use classes.

^c^ Adjusted for sex, maternal education, emotional and behavioral problems (Strengths and Difficulties Questionnaire score at age 9 years), and maternal cigarette smoking during pregnancy.

When comparing the substance use classes with each other (eTable 7 in the Supplement), there was strong evidence to rule out equivalence between the association of late-onset cannabis use and late-onset cigarette-only use with psychotic experiences (OR, 3.63; 95% CI, 1.12-11.76). There was insufficient evidence to support a difference between the association of early-onset cannabis and early-onset cigarette-only use with psychotic experiences, although this was based on smaller numbers, or to support a difference between late-onset and early-onset cannabis use classes. Results were similar when excluding individuals with psychotic experiences at age 12 years (eTable 7 in the Supplement).

### Sensitivity Analyses

Results of associations between class membership and subsequent psychotic experiences were substantively the same when excluding participants whose psychotic experiences only ever occurred within 2 hours of any drug use (eTable 8 in the Supplement) and when examining narrower or broader psychotic outcome definitions (eTable 9 in the Supplement).

### Psychotic Experiences at Age 12 Years and Patterns of Cigarette and/or Cannabis Use at Ages 14 to 19 Years 

Definite psychotic experiences at age 12 years were associated with increased odds of subsequent late-onset cigarette-only use (OR, 1.76; 95% CI, 1.01-3.10) and late-onset cannabis use (OR, 1.66; 95% CI, 0.94-2.91) as compared with nonusers (Table 3 and eTable 10 in the Supplement).

There was little evidence that psychotic experiences at age 12 years were associated with increased odds of early-onset cigarette-only or cannabis use; however, these classes had smaller membership (Figure and Table 3). Adjusting for confounders had minimal impact on associations between psychotic experiences at age 12 years and classes of subsequent cannabis and/or cigarette use. The effect estimates for all classes were smaller, and evidence of association weaker (particularly for early-onset classes), when restricting the analysis to nonusers of cigarettes and/or cannabis at age 12 years (eTable 11 in the Supplement).

## Discussion

Both early-onset and late-onset cannabis use classes were associated with psychotic experiences at age 18 years and were only minimally attenuated after adjusting for potential confounders. In contrast, there was inadequate evidence to support an association between either early-onset or late-onset cigarette-only use and psychotic experiences in the adjusted analyses. There was also evidence that participants in the late-onset cannabis use class had higher odds of psychotic experiences than those in the late-onset cigarette-only use class, the 2 most common substance use classes in our data. There was no evidence to support a stronger association of early-onset cannabis use compared with late-onset cannabis use on psychotic outcomes as proposed by some, but not all, studies,^1^ although the relatively small size of the early-onset class has limited power to detect small-moderate effects.

Adjusting for a broad range of potential confounders did not alter the estimate of association for either the early- or late-onset cannabis use class but resulted in an approximately 60% attenuation of the estimate for the early-onset cigarette-only class. This difference in the impact of adjustment for confounders indicates that the association between cannabis use and psychotic experiences is more robust against explanations of residual confounding than that for tobacco use.

In comparison, we found little evidence that psychotic experiences in childhood led to increased cannabis use. As other observational studies have indicated,^28,29,30^ the self-medication hypothesis does not appear to adequately explain the association between cannabis use and psychosis. Such a relationship for tobacco use is also not well supported by our data.

The uncertainty around our estimates means we cannot exclude a possible association of cigarette-only use with psychotic experiences. A number of longitudinal studies have reported that tobacco users are at greater risk for later psychotic disorders.^2,3,4,5,31,32^ However, none of these studies adjusted for cannabis use, and while adjusting for diagnoses of drug abuse in 2 of the studies substantially attenuated associations for cigarette smoking^4^ or snus use,^32^ this is likely a poor measure of cannabis use and hence may have underestimated its confounding effect. In the only longitudinal study, to our knowledge, that has adjusted for cannabis use, this substantially attenuated the association for cigarette smoking, with the fully adjusted model supporting a protective effect of smoking on schizophrenia.^33^

In our previous study using the Avon Longitudinal Study of Parents and Children cohort, we reported that the association between cannabis use and psychotic experiences was altered only slightly by adjusting for early or childhood confounders but that interpretation of results adjusted for tobacco use was problematic because of a strong relationship between these measures.^16^ In the current study, we are better able to disentangle differential effects of tobacco use from those of cannabis use through use of data at multiple time points to describe patterns of use associated with both of these substances over time. Our findings are consistent with another study in which adjusting for confounding using fixed-effects regression to deal with unmeasured time-invariant effects resulted in much greater attenuation of association between cigarette smoking and psychotic symptoms than for cannabis use.^8^

Another approach to strengthen causal inference is mendelian randomization whereby genetic variants act as assumed unconfounded proxy measures for exposure status.^34^ One study reported weak evidence of association between a genetic variant within the *CHRNA5-A3-B4* gene cluster and being prescribed antipsychotic medication.^35^ However, despite this association being stronger in smokers than nonsmokers (as would be expected if this was due to a causal role of smoking on psychosis), there was little statistical evidence for this (*P* = .60).^35^

We recently conducted a mendelian randomization study and found little association between cigarette smoking initiation and schizophrenia risk,^36^ while our mendelian randomization study of cannabis initiation and schizophrenia risk provided evidence for causal pathways operating in both directions.^14^ However, in both cases our analyses were restricted to smoking and cannabis initiation and might not reflect the effects of longer-term regular use. The lack of adequate samples and strong genetic instruments for regular cannabis use limit current use of mendelian randomization studies to further inform causal inference.

### Limitations

One of the strengths of our study is that we use a large, well-characterized cohort, albeit of mostly European ancestry, with multiple measures of exposures of interest and psychotic experience data over time, with data on a broad range of potential confounders collected prospectively. Using information across the entire adolescent period rather than from a single time point means our results are much less prone to measurement error. However, there is considerable attrition over time, although the use of a latent class method with longitudinal data allows us to maximize use of data for individuals even where participation and question response have been sporadic, and hence to minimize potential selection bias to some extent. While use of a latent class method confers a number of advantages over using measures at single time points, it was not possible to define a class of individuals who use cannabis without tobacco as most cannabis users smoke cannabis in combination with tobacco.^37^ Therefore, we cannot rule out whether the associations observed between the cannabis use class and psychotic experiences are exacerbated by the combined use of cannabis and cigarettes. While experimental studies of intravenous Δ9-THC support a causal effect of cannabis on acute psychotic experiences in the absence of tobacco,^10^ there is some evidence that smoking cannabis with tobacco also increases the amount of THC inhaled per gram.^38^

Furthermore, we have previously found that a substantial proportion of people who smoke cigarettes most heavily also use cannabis, and thus the cigarette-only class might not include those who have been most heavily exposed to tobacco. As the cannabis use group in our study included occasional (1-3 times in the past 6 months) and frequent (daily) users, we were unable to differentiate whether our findings are mainly driven by frequent users; including frequency of substance use data resulted in an unstable model. Our study was also not able to examine associations of longer-term cumulative cannabis and tobacco use and psychosis outcomes, although these analyses may become tractable in the future.

While psychotic experiences in the population are relatively poor predictors of psychotic disorder,^23^ they represent the key characteristic of such disorders, and understanding their etiology almost certainly has relevance to understanding the etiology of clinically defined psychosis. However, we were not adequately powered to investigate the effects of cannabis or cigarette use on psychotic disorders and cannot rule out different effects of these substances on other psychosis-related psychopathology, such as negative symptoms. We were also unable to tease out associations of cannabis with chronic vs acute psychotic outcomes, although excluding individuals who reported psychotic experiences only ever occurring within 2 hours of using drugs had minimal effect on our results. Nevertheless, given the long half-life of THC, the only way of determining whether cannabis use can lead to chronic psychotic disorders that persist long after potential effects of exogenous cannabinoids is to study regular users of cannabis who subsequently become abstinent.^30^

The one longitudinal study of which we are aware that examined this relationship reported only weak evidence of association between ex–cannabis use and psychotic experiences, although there were relatively few ex–cannabis users.^30^ Given the age of the participants over the course of our study, we were not able to identify a class of ex–cannabis users to clarify this relationship; however, long-term follow-up of this cohort may enable us to address this question more robustly.

## Conclusions

Our study found that both adolescent cannabis use and cigarette use are associated with increased risk for subsequent psychotic experiences. This association was greater for cannabis. Associations observed between tobacco use and psychotic experiences are more likely than those for cannabis use to be influenced by other characteristics of people who develop psychotic experiences.

## Notes

Supplement. **eAppendix.** **eTable 1.** Proportion of Individuals Who Class Themselves as Cannabis Users, But Not Cigarette Users, That Smoke Cannabis With Tobacco **eTable 2.** Comparison of Class Proportions Between Six and Four Time Point Models **eTable 3.** Unadjusted and Adjusted Associations Between Cigarette and/or Cannabis Use and Psychotic Experiences at Age 18 Years, Individually Adjusting for Each Potential Confounder and Adjusting for All Confounders **eTable 4.** Category and Missingness Frequencies and Percentages Across Data Collection Time Points **eTable 5.** Sociodemographic Differences Between Individuals Who Were Included in the Derivation of Cannabis/Cigarette Use Latent Classes and Those Who Were Missing From the Analysis **eTable 6.** Longitudinal Latent Class Model Fit Statistics Based on 6 Repeated Measures **eTable 7.** Unadjusted and Adjusted Associations Between Cigarette and/or Cannabis Use and Psychotic Experiences at Age 18 Years **eTable 8.** Associations Between Cigarette and/or Cannabis Use and Psychotic Experiences Not Experienced Within 2 Hours of Drug or Alcohol Use at Age 18 Years **eTable 9.** Associations Between Cigarette/Cannabis Use and Psychotic Experiences vs Remainder and Psychotic Disorder vs Remainder at Age 18 Years **eTable 10.** Unadjusted and Adjusted Associations Between Psychotic Experiences at Age 12 Years and Subsequent Cigarette and/or Cannabis Use **eTable 11.** Associations Between Psychotic Experiences at Age 12 Years and Subsequent Cigarette and/or Cannabis Use, Limiting to Non-Users at Age 12 Years **eFigure.** Comparisons Between 5-Class Models of Cigarette/Cannabis Use Patterns Created Using Six and Four Time Points **eReferences.**
