# Supplementary material for: Association of Combined Patterns of Tobacco and Cannabis Use in Adolescence With Psychotic Experiences
Source: JAMA Psychiatry. 2018 Jan 17;75(3):240–6. doi: 10.1001/jamapsychiatry.2017.4271 (PMC5885944; doi:10.1001/jamapsychiatry.2017.4271)
Supplement: Supplement. — eAppendix. eTable 1. Proportion of Individuals Who Class Themselves as Cannabis Users, But Not Cigarette Users, That Smoke Cannabis With Tobacco eTable 2. Comparison of Class Proportions Between Six and Four Time Point Models eTable 3. Unadjusted and Adjusted Associations Between Cigarette and/or Cannabis Use and Psychotic Experiences at Age 18 Years, Individually Adjusting for Each Potential Confounder and Adjusting for All Confounders eTable 4. Category and Missingness Frequencies and Percentages Across Data Collection Time Points eTable 5. Sociodemographic Differences Between Individuals Who Were Included in the Derivation of Cannabis/Cigarette Use Latent Classes and Those Who Were Missing From the Analysis eTable 6. Longitudinal Latent Class Model Fit Statistics Based on 6 Repeated Measures eTable 7. Unadjusted and Adjusted Associations Between Cigarette and/or Cannabis Use and Psychotic Experiences at Age 18 Years eTable 8. Associations Between Cigarette and/or Cannabis Use and Psychotic Experiences Not Experienced Within 2 Hours of Drug or Alcohol Use at Age 18 Years eTable 9. Associations Between Cigarette/Cannabis Use and Psychotic Experiences vs Remainder and Psychotic Disorder vs Remainder at Age 18 Years eTable 10. Unadjusted and Adjusted Associations Between Psychotic Experiences at Age 12 Years and Subsequent Cigarette and/or Cannabis Use eTable 11. Associations Between Psychotic Experiences at Age 12 Years and Subsequent Cigarette and/or Cannabis Use, Limiting to Non-Users at Age 12 Years eFigure. Comparisons Between 5-Class Models of Cigarette/Cannabis Use Patterns Created Using Six and Four Time Points eReferences. [file jamapsychiatry-75-240-s001.pdf]

Supplementary Online Content

Jones HJ, Gage SH, Heron J, et al. Association of combined patterns of tobacco and cannabis use in adolescence with psychotic experiences. *JAMA Psychol*. Published online January 17, 2018. doi:10.1001/jamapsychiatry.2017.4271

eAppendix.

eTable 1. Proportion of Individuals Who Class Themselves as Cannabis Users, But Not Cigarette Users, That Smoke Cannabis With Tobacco

eTable 2. Comparison of Class Proportions Between Six and Four Time Point Models

eTable 3. Unadjusted and Adjusted Associations Between Cigarette and/or Cannabis Use and Psychotic Experiences at Age 18 Years, Individually Adjusting for Each Potential Confounder and Adjusting for All Confounders

eTable 4. Category and Missingness Frequencies and Percentages Across Data Collection Time Points

eTable 5. Sociodemographic Differences Between Individuals Who Were Included in the Derivation of Cannabis/Cigarette Use Latent Classes and Those Who Were Missing From the Analysis

eTable 6. Longitudinal Latent Class Model Fit Statistics Based on 6 Repeated Measures

eTable 7. Unadjusted and Adjusted Associations Between Cigarette and/or Cannabis Use and Psychotic Experiences at Age 18 Years

eTable 8. Associations Between Cigarette and/or Cannabis Use and Psychotic Experiences Not Experienced Within 2 Hours of Drug or Alcohol Use at Age 18 Years

eTable 9. Associations Between Cigarette/Cannabis Use and Psychotic Experiences vs Remainder and Psychotic Disorder vs Remainder at Age 18 Years

eTable 10. Unadjusted and Adjusted Associations Between Psychotic Experiences at Age 12 Years and Subsequent Cigarette and/or Cannabis Use

eTable 11. Associations Between Psychotic Experiences at Age 12 Years and Subsequent Cigarette and/or Cannabis Use, Limiting to Non-Users at Age 12 Years

eFigure. Comparisons Between 5-Class Models of Cigarette/Cannabis Use Patterns Created Using Six and Four Time Points

eReferences

This supplementary material has been provided by the authors to give readers additional information about their work.

© 2017 Jones HJ et al. *JAMA Psychiatry.*

eAppendix

Repeated measures of cigarette and/or cannabis use

Measures taken at approximate age 14 years, 16 years and 18 years were collected as part of ALSPAC assessment clinics using a computerised interview. Measures taken at approximate age 15 years, 17 years and 19 years were collected via ALSPAC postal questionnaires. For each time point, individuals were deemed as cigarette users if they were current smokers who smoked at least 1-3 in the previous 6 months (age 14 years), who smoked less that once a week, weekly or daily (age 15 and 16 years), had smoked less that once a week, weekly or daily in the last 30 days (age 16, 18 and 19 years). Individuals were deemed as non-cigarette users if they had never smoked a cigarette, if they had only tried cigarettes once or twice (age 15 and 17 years) or if they had not smoked in the last 6 months (age 14 years) or last 30 days (age 16, 18 and 19 years). For each time point, individuals were deemed as cannabis users if they had used or taken cannabis at least 1-3 times in the past 6 months (age 14 years), currently take cannabis less than weekly, weekly or daily (age 15, 16 and 17 years) or at least monthly or less in the last 12 months (age 18 and 19 years). Individuals were deemed as non-cannabis users if they had never tried cannabis, if they had only ever tried cannabis once or twice or if they used to sometimes use or take cannabis but had since stopped.

Psychotic experiences

The semi-structured Psychosis-Like Symptom Interview (PLIKSi)[1,2] was used as part of ALSPAC assessment clinics to assess psychotic experiences. The interviewers were psychology graduates trained in assessment using the SCAN psychosis section and using the PLIKSi. Psychotic experiences were rated as not present, suspected, or definitely psychotic. Unclear responses were always ‘rated down’ and symptoms were rated as definite only when a clear example was provided. At regular intervals, samples of recorded interviews were also rated by a psychiatrist to ensure that interviewers were rating experiences correctly. The PLIKSi shows very good inter-rater and test-retest reliability.[3]

The primary psychotic experience measures at ages 12 and 18 years were binary variables relating to whether an individual had at least one definite psychotic experience compared to suspected or no psychotic experiences.

Secondary outcome measures at ages 12 and 18 years were binary variables relating to whether an individual had at least one definite psychotic experience compared to no psychotic experiences. At age 18 years, an additional variable was created and tested relating to whether or not an individual had a psychotic disorder. Cases of psychotic disorder were defined as individuals with definite psychotic experiences that were not attributable to the effects of sleep or fever and when the psychotic experiences: i) occurred at least once per month over the past 6 months, and ii) caused severe distress, had a very negative effect on social/occupational function, or led to help seeking from a professional source.[2]

© 2017 Jones HJ et al. *JAMA Psychiatry.*

Potential confounders

A number of potential confounders were examined: sex (binary measure), family history of schizophrenia or depression (binary measure assessed via maternal and paternal questionnaire), family history of drug use (binary measure assessed via maternal and paternal questionnaire), maternal smoking during pregnancy (binary measure assessed via maternal questionnaire), paternal smoking during pregnancy (binary measure assessed via maternal and paternal questionnaire), maternal education (5-level categorical measure assessed via maternal questionnaire), maternal/paternal social class (4-level categorical measure assessed via maternal questionnaire), IQ at age 8 years (assessed via the Wechsler Intelligence Scale for Children[4]), bullying at age 8 years (a binary measure relating to whether individual experienced relational or overt bullying, assessed via interview at age 8 years), Strengths and Difficulties Questionnaire (SDQ)[5] score at age 9 years relating to conduct problems, hyperactivity, emotional symptoms, peer problems and prosocial behaviour (total score assessed via parental-completed questionnaire when child was age 9 years), alcohol use at age 12 years (ordinal measure: none/less than weekly/weekly consumption of at least one whole drink assessed via child-completed interview), exposure to traumatic events (ordinal measure: none/moderate/severe) in mid-childhood (81 and 103 months) (assessed via a parental-completed questionnaire; see Crane et al. 2014[6] for more detail).Association analyses

Multinomial regression was used to assess whether psychotic experiences at age 12 years were associated with subsequent latent class membership. Associations were assessed using a manual implementation of the bias-adjusted three-step method (see Heron et al. 2015[7] for more detail and example of code). The latent classes were first derived without the presence of psychotic experiences. The resulting logit parameters defining the relationship between modal and latent classes were used as constraints allowing odds ratios and confidence intervals for the associations to be calculated without influencing latent class membership.

© 2017 Jones HJ et al. *JAMA Psychiatry.*

eTable 1. Proportion of individuals who class themselves as cannabis users, but not cigarette users, that smoke cannabis with tobacco

Age (years)	No. cannabis users but not cigarette users	No. (%) use cannabis with tobacco
**16**	110	90 (82)
**17**	100	90 (90)
**18**	197	164 (83)

Note: Data on methods of cannabis use are not available at the other ages used within the current study.

© 2017 Jones HJ et al. *JAMA Psychiatry.*

eTable 2. Comparison of class proportions between six and four time point models

		**Number of time points in model**	
		**6**	**4**
**Class**	**Early-onset cannabis with/without cigarette use**	0.03	0.03
	**Late-onset cannabis with/without cigarette use**	0.12	0.08
	**Early-onset cigarette-only use**	0.04	0.03
	**Late-onset cigarette-only use**	0.15	0.15
	**Non-use**	0.66	0.71

© 2017 Jones HJ et al. *JAMA Psychiatry.*

eTable 3. Unadjusted and adjusted associations between cigarette and/or cannabis use and psychotic experiences at age 18 years, individually adjusting for each potential confounder and adjusting for all confounders

		Definite PE			
		Unadjusted		Adjusted	
	N	OR (95% CI)[a]	*P* value	OR (95% CI)[a]	*P* value
**Sex**					
Early-onset cigarette-only	3854	2.58 (1.02, 6.51)	<0.001	2.05 (0.79, 5.35)	<0.001
Early-onset cannabis		3.28 (1.48, 7.26)		3.40 (1.56, 7.44)	
Late-onset cigarette-only		0.90 (0.36, 2.24)		0.75 (0.29, 1.95)	
Late-onset cannabis		3.47 (2.09, 5.76)		3.42 (2.06, 5.69)	
**Family history of schizophrenia or depression**					
Early-onset cigarette-only	2950	2.80 (0.92, 8.54)	<0.001	2.73 (0.90, 8.33)	<0.001
Early-onset cannabis		4.82 (2.16, 10.72)		4.78 (2.14, 10.68)	
Late-onset cigarette-only		0.93 (0.33, 2.68)		0.96 (0.34, 2.70)	
Late-onset cannabis		3.53 (2.00, 6.21)		3.46 (1.97, 6.10)	
**Family history of drug use**					
Early-onset cigarette-only	2950	2.72 (0.86, 8.55)	<0.001	2.72 (0.86, 8.55)	<0.001
Early-onset cannabis		4.80 (2.15, 10.70)		4.80 (2.15, 10.70)	
Late-onset cigarette-only		0.93 (0.32, 2.68)		0.93 (0.32, 2.68)	
Late-onset cannabis		3.54 (2.01, 6.23)		3.54 (2.01, 6.23)	
**Maternal smoking during pregnancy**					
Early-onset cigarette-only	3770	2.70 (1.07, 6.81)	<0.001	2.16 (0.82, 5.70)	<0.001
Early-onset cannabis		3.38 (1.53, 7.48)		3.06 (1.38, 6.79)	
Late-onset cigarette-only		0.86 (0.32, 2.27)		0.85 (0.33, 2.19)	
Late-onset cannabis		3.65 (2.19, 6.08)		3.41 (2.05, 5.68)	

© 2017 Jones HJ et al. *JAMA Psychiatry.*

**Paternal smoking during pregnancy**†					
Early-onset cigarette-only	3030	1.79 (0.50, 6.48)	<0.001	1.47 (0.38, 5.7)	<0.001
Early-onset cannabis		4.09 (1.86, 8.97)		3.68 (1.66, 8.13)	
Late-onset cigarette-only		0.95 (0.36, 2.54)		0.94 (0.36, 2.43)	
Late-onset cannabis		3.63 (2.09, 6.30)		3.29 (1.85, 5.83)	
**Maternal education**					
Early-onset cigarette-only	3767	2.31 (0.84, 6.33)	<0.001	1.94 (0.64, 5.90)	<0.001
Early-onset cannabis		3.28 (1.50, 7.18)		3.65 (1.67, 7.98)	
Late-onset cigarette-only		0.86 (0.34, 2.19)		0.83 (0.33, 2.11)	
Late-onset cannabis		3.36 (2.01, 5.61)		3.43 (2.05, 5.76)	
**Highest parental social class**†					
Early-onset cigarette-only	3635	2.44 (0.90, 6.58)	<0.001	2.12 (0.73, 6.18)	<0.001
Early-onset cannabis		3.22 (1.40, 7.40)		3.42 (1.49, 7.86)	
Late-onset cigarette-only		0.84 (0.31, 2.25)		0.79 (0.29, 2.15)	
Late-onset cannabis		3.51 (2.08, 5.92)		3.57 (2.12, 6.03)	
**IQ at age 8 years**					
Early-onset cigarette-only	3464	2.80 (1.04, 7.59)	<0.001	2.73 (1.00, 7.42)	<0.001
Early-onset cannabis		3.80 (1.73, 8.36)		3.90 (1.78, 8.56)	
Late-onset cigarette-only		0.87 (0.32, 2.32)		0.86 (0.32, 2.30)	
Late-onset cannabis		3.06 (1.75, 5.38)		3.10 (1.77, 5.43)	
**Experiencing bullying at age 8 years**					
Early-onset cigarette-only	3373	2.86 (1.06, 7.71)	<0.001	2.79 (1.03, 7.55)	<0.001
Early-onset cannabis		3.53 (1.53, 8.16)		3.54 (1.52, 8.28)	
Late-onset cigarette-only		0.85 (0.31, 2.35)		0.85 (0.31, 2.32)	
Late-onset		3.40 (1.96,		3.40 (1.96,	

© 2017 Jones HJ et al. *JAMA Psychiatry*.

cannabis		5.88)		5.89)	
**Emotional/behavioural problems (SDQ score age 9 years)**					
Early-onset cigarette-only	3424	2.75 (1.02, 7.36)	<0.001	2.44 (0.86, 6.98)	<0.001
Early-onset cannabis		3.78 (1.72, 8.31)		3.67 (1.64, 8.17)	
Late-onset cigarette-only		0.91 (0.36, 2.34)		0.94 (0.37, 2.36)	
Late-onset cannabis		2.95 (1.63, 5.33)		2.95 (1.64, 5.32)	
**Alcohol use at age 12 years**					
Early-onset cigarette-only	3377	2.49 (0.92, 6.71)	<0.001	2.42 (0.88, 6.65)	<0.001
Early-onset cannabis		3.06 (1.34, 7.02)		2.87 (1.22, 6.74)	
Late-onset cigarette-only		0.85 (0.33, 2.23)		0.88 (0.35, 2.21)	
Late-onset cannabis		3.16 (1.86, 5.38)		3.06 (1.8, 5.19)	
**Trauma during mid childhood**					
Early-onset cigarette-only	3592	2.63 (0.97, 7.13)	<0.001	2.59 (0.95, 7.07)	<0.001
Early-onset cannabis		3.7 (1.68, 8.14)		3.63 (1.66, 7.97)	
Late-onset cigarette-only		0.8 (0.29, 2.18)		0.8 (0.29, 2.18)	
Late-onset cannabis		3.03 (1.76, 5.24)		3.06 (1.77, 5.28)	
**All potential confounders**					
Early-onset cigarette-only	1854	1.81 (0.16, 20.83)	<0.001	0.78 (0.03, 19.92)	0.01
Early-onset cannabis		6.19 (2.42, 15.86)		4.89 (1.77, 13.53)	
Late-onset cigarette-only		1.35 (0.44, 4.09)		1.12 (0.36, 3.48)	
Late-onset cannabis		2.97 (1.31, 6.77)		2.77 (1.16, 6.64)	

Note: PE, psychotic experiences; OR, odds ratio; 95% CI, 95% confidence interval; *P* value, omnibus *P* value for association between cigarette/cannabis use classes and psychotic experiences at age 18 years; SDQ, Strengths and Difficulties Questionnaire.

[a] Compared to non-use class.

[†] Adjusting for paternal smoking during pregnancy and parental social class made little difference when already accounting for maternal education and maternal smoking during pregnancy, therefore these variables were omitted from the analysis in the main paper

eTable 4. Category and missingness frequencies and percentages across data collection time points

Age (years)	Category	Frequency	Percentage	Percentage among responders
14	Non-users	4,449	83.94	95.60
	Cigarette-only users	68	1.28	1.46
	Cannabis with/without cigarette users	137	2.58	2.94
	Missing	646	12.19	-
15	Non-users	4,273	80.62	94.18
	Cigarette-only users	164	3.09	3.61
	Cannabis with/without cigarette users	100	1.89	2.20
	Missing	763	14.40	-
16	Non-users	3,614	68.19	81.75
	Cigarette-only users	412	7.77	9.32
	Cannabis with/without cigarette users	395	7.45	8.93
	Missing	879	16.58	-
17	Non-users	3,273	61.75	78.51
	Cigarette-only users	487	9.19	11.68
	Cannabis with/without cigarette users	409	7.72	9.81
	Missing	1,131	21.34	-
18	Non-users	2,385	45.00	67.35
	Cigarette-only users	477	9.00	13.47
	Cannabis with/without cigarette users	679	12.81	19.18
	Missing	1,759	33.19	-
19	Non-users	1,998	37.70	68.26
	Cigarette-only users	452	8.53	15.44
	Cannabis with/without cigarette users	477	9.00	16.30
	Missing	2,373	44.77	-

© 2017 Jones HJ et al. *JAMA Psychiatry.*

eTable 5. Sociodemographic differences between individuals who were included in the derivation of cannabis/cigarette use latent classes and those who were missing from the analysis

			Included in the derivation of cannabis/cigarette use latent classes?		
**Measure**			**No**	**Yes**	***P***
**Sex**	**male**	**N (%)**	5,309 (55.55)	2,326 (43.92)	<0.001
	**female**	**N (%)**	4,249 (44.45)	2,970 (56.08)	
**Highest parental social class**	**professional/managerial**	**N (%)**	3,122 (47.98)	3,206 (64.39)	<0.001
	**non-professional/managerial**	**N (%)**	3,385 (52.02)	1,773 (35.61)	
**Household income (quintiles; lowest income = 1, highest income = 5)**	**1**	**N (%)**	1,338 (26.12)	651 (13.55)	<0.001
	**2**	**N (%)**	1,102 (21.51)	866 (18.02)	
	**3**	**N (%)**	1,002 (19.56)	974 (20.27)	
	**4**	**N (%)**	886 (17.29)	1,100 (22.89)	
	**5**	**N (%)**	795 (15.52)	1,214 (25.27)	
**Crowding index**	**≤ 1 individual per room**	**N (%)**	7,391 (90.62)	4,948 (96.92)	<0.001
	**> 1 individual per room**	**N (%)**	765 (9.38)	157 (3.08)	
**Parity**	**0**	**N (%)**	3,342 (41.91)	2531 (49.15)	<0.001
	**1**	**N (%)**	2,788 (34.96)	1,800 (34.95)	
	**2**	**N (%)**	1,262 (15.83)	619 (12.02)	
	**3+**	**N (%)**	582 (7.30)	200 (3.88)	
**Highest maternal education qualification**	**CSE**	**N (%)**	1,953 (26.65)	574 (11.11)	<0.001
	**Vocational**	**N (%)**	847 (11.56)	382 (7.40)	
	**O level**	**N (%)**	2,566 (35.02)	1,761 (34.09)	

© 2017 Jones HJ et al. *JAMA Psychiatry.*

	**A level**	**N (%)**	1,356 (18.50)	1,445 (27.98)	
	**Degree**	**N (%)**	606 (8.27)	1,003 (19.42)	
**Ethnicity**	**non-white**	**N (%)**	232 (3.20)	94 (1.82)	<0.001
	**white**	**N (%)**	7,018 (96.80)	5,057 (98.18)	
**Birthweight (g)**		**N**	8,664	5,231	<0.001
		**Mean**	3361.08	3414.93	
		**SD**	604.51	537.82	

Note: CSE, Certificate of Secondary Education; SD, standard deviation; *P*, *P* value for chi-squared test (categorical measures) or t-test (birthweight) of differences between individuals used to derive cannabis/cigarette use latent classes and those who were missing from the analysis.

© 2017 Jones HJ et al. *JAMA Psychiatry.*

eTable 6. Longitudinal latent class model fit statistics based on 6 repeated measures

No. Classes	SSABIC	Entropy	Smallest class (%)	LMR-LRT
**Complete case (n = 1,458)**				
1	8568.38	n/a	100	n/a
2	7045.66	0.895	24.60	<0.001
3	6913.16	0.879	11.36	0.04
4	6837.96	0.902	4.38	0.85
5	6815.46	0.879	1.99	0.18
6	6826.22†	0.886	0.69	0.72[†]
**Data present for 3 or more time points (n = 5,300)**				
1	26251.01	n/a	100	n/a
2	22299.46	0.81	26.18	<0.001
3	21897.66	0.79	11.58	<0.001
4	21667.89	0.78	4.99	0.01
5	21601.50	0.77	3.22	0.05
6	21606.46†	0.77	1.79	1.00[†]

Note: SSABIC, sample size adjusted Bayesian Information Criterion, lower levels of SSABIC suggest a better model fit; LMR-LRT, Lo-Mendell-Rubin likelihood ratio test, LMR-LRT tests for an improvement in fit between k-1 and the k class models.[8]

[†] Note that the SSABIC and LMR-LRT provided no strong evidence that a 6-class model was an improvement on a 5-class model.

© 2017 Jones HJ et al. *JAMA Psychiatry.*

eTable 7. Unadjusted and adjusted associations between cigarette and/or cannabis use and psychotic experiences at age 18 years

	**All available data (n = 3328, 4.27% definite PE)**				**Limiting to individuals without PE at 12 years of age (n = 3179, 3.52% definite PE)**			
	**Unadjusted**		**Adjusted**†		**Unadjusted**		**Adjusted**†	
**Exposure**[a]	**OR (95% CI)**	***P***	**OR (95% CI)**	***P***	**OR (95% CI)**	***P***	**OR (95% CI)**	***P***
Early cigarette-only users	3.03 (1.13, 8.14)	<0.001	1.78 (0.54, 5.88)	<0.001	3.36 (1.18, 9.61)	<0.001	2.11 (0.63, 7.05)	<0.001
Early cannabis with/without cigarette users	3.79 (1.73, 8.31)		3.70 (1.66, 8.25)		3.48 (1.35, 8.98)		3.39 (1.31, 8.79)	
Late cigarette-only users	0.84 (0.31, 2.31)		0.73 (0.27, 1.98)		0.95 (0.30, 2.99)		0.84 (0.27, 2.60)	
Late cannabis with/without cigarette users	3.05 (1.69, 5.53)		2.97 (1.63, 5.40)		3.91 (2.11, 7.25)		3.73 (2.00, 6.94)	
Early cigarette-only users versus Early cannabis with/without cigarette users	0.80 (0.22, 2.94)		0.48 (0.11, 2.16)		0.96 (0.23, 4.13)		0.62 (0.13, 3.07)	
Late cigarette-only users versus Early cannabis with/without cigarette users	0.22 (0.07, 0.70)		0.20 (0.06, 0.63)		0.27 (0.07, 1.04)		0.25 (0.06, 0.94)	
Late cannabis with/without cigarette users versus Early cannabis with/without cigarette users	0.81 (0.30, 2.15)		0.80 (0.29, 2.18)		1.12 (0.37, 3.43)		1.10 (0.36, 3.37)	
Late cannabis with/without cigarette users versus Late cigarette-	3.63 (1.12, 11.76)		4.06 (1.25, 13.16)		4.12 (1.13, 14.96)		4.45 (1.24, 15.99)	

© 2017 Jones HJ et al. *JAMA Psychiatry.*

only users								

Note: PE, psychotic experiences; OR, odds ratio; 95% CI, 95% confidence interval; UCI, upper 95% confidence interval; *P*, omnibus *P* value for association between cigarette/cannabis use classes and psychotic experiences at age 18 years.

[a] Compared to non-use class unless otherwise stated.

† Adjusted for sex, maternal education, emotional/behavioural problems (Strengths and Difficulties Questionnaire (SDQ) score age 9 years) and maternal cigarette smoking during pregnancy.

© 2017 Jones HJ et al. *JAMA Psychiatry.*

eTable 8. Associations between cigarette and/or cannabis use and psychotic experiences not experienced within 2 hours of drug or alcohol use at age 18 years

	**All available data (n = 3819, 4.01% definite PE)**		**Limiting to individuals without PE at 12 years of age (n = 3646, 3.32% definite PE)**	
**Exposure[a]**	**OR (95% CI)**	***P***	**OR (95% CI)**	***P***
Early cigarette-only users	2.97 (1.24, 7.11)	<0.001	3.24 (1.29, 8.12)	0.001
Early cannabis with/without cigarette users	1.97 (0.69, 5.65)		1.59 (0.40, 6.32)	
Late cigarette-only users	0.94 (0.39, 2.27)		0.79 (0.25, 2.56)	
Late cannabis with/without cigarette users	2.62 (1.46, 4.72)		3.43 (1.89, 6.22)	
Early cigarette-only users versus Early cannabis with/without cigarette users	1.51 (0.36, 6.29)		2.03 (0.36, 11.47)	
Late cigarette-only users versus Early cannabis with/without cigarette users	0.48 (0.13, 1.69)		0.50 (0.09, 2.69)	
Late cannabis with/without cigarette users versus Early cannabis with/without cigarette users	1.33 (0.39, 4.5)		2.15 (0.48, 9.71)	
Late cannabis with/without cigarette users versus Late cigarette-only	2.80 (0.97, 8.07)		4.31 (1.15, 16.14)	

© 2017 Jones HJ et al. *JAMA Psychiatry.*

users				

Note: PE, psychotic experiences; OR, odds ratio; 95% CI, 95% confidence interval; *P*, omnibus *P* value for association between cigarette/cannabis use classes and psychotic experiences not experienced within 2 hours of drug or alcohol use at age 18 years.

[a] Compared to non-use class unless otherwise stated.

© 2017 Jones HJ et al. *JAMA Psychiatry.*

eTable 9. Associations between cigarette/cannabis use and psychotic experiences versus remainder and psychotic disorder versus remainder at age 18 years

	All available data				Limiting to individuals without PE at 12 years of age			
	Definite/suspected PE versus remainder (n = 3855, 8.53% PE)		Disorder versus remainder (n = 3855, 1.45% disorder)		Definite/suspected PE versus remainder (n = 3676, 7.43% PE)		Disorder versus remainder (n = 3676, 1.09% disorder)	
**Exposure[a]**	**OR (95% CI)**	***P***	**OR (95% CI)**	***P***	**OR (95% CI)**	***P***	**OR (95% CI)**	***P***
Early cigarette-only users	2.64 (1.32, 5.30)	<0.001	3.14 (0.75, 13.22)	0.02	2.94 (1.45, 5.97)	<0.0001	2.52 (0.46, 13.81)	0.31
Early cannabis with/without cigarette users	3.77 (2.09, 6.79)		4.20 (1.26, 14.01)		4.01 (2.12, 7.62)		2.56 (0.46, 14.1)	
Late cigarette-only users	1.02 (0.57, 1.84)		1.40 (0.40, 4.89)		0.96 (0.48, 1.92)		0.92 (0.16, 5.18)	
Late cannabis with/without cigarette users	2.57 (1.66, 3.96)		2.42 (0.87, 6.75)		2.96 (1.88, 4.67)		2.28 (0.72, 7.23)	
Early cigarette-only users versus Early cannabis with/without cigarette users	0.70 (0.27, 1.81)		0.75 (0.11, 5.07)		0.73 (0.27, 1.97)		0.99 (0.08, 12.01)	
Late cigarette-only users versus Early cannabis	0.27 (0.13, 0.58)		0.33 (0.07, 1.52)		0.24 (0.10, 0.56)		0.36 (0.04, 3.30)	

© 2017 Jones HJ et al. *JAMA Psychiatry.*

with/without cigarette users								
Late cannabis with/without cigarette users versus Early cannabis with/without cigarette users	0.68 (0.33, 1.43)		0.58 (0.12, 2.78)		0.74 (0.33, 1.63)		0.89 (0.11, 7.13)	
Late cannabis with/without cigarette users versus Late cigarette-only users	2.50 (1.21, 5.17)		1.73 (0.36, 8.25)		3.10 (1.35, 7.09)		2.49 (0.31, 20.15)	

Note: PE, psychotic experiences; OR, odds ratio; 95% CI, 95% confidence interval; *P*, omnibus *P* value for association between cigarette/cannabis use and psychotic experiences/disorder at age 18 years.

[a] Compared to non-use class unless otherwise stated.

© 2017 Jones HJ et al. *JAMA Psychiatry.*

eTable 10. Unadjusted and adjusted associations between psychotic experiences at age 12 years and subsequent cigarette and/or cannabis use

Exposure	Early-onset cigarette	Early-onset cannabis	Late-onset cigarette	Late-onset cannabis	Early-onset cigarette versus Early-onset cannabis	Late-onset cigarette versus Early-onset cannabis	Late-onset cannabis versus Early-onset cannabis	Late-onset cannabis versus Late-onset cigarette	
	**OR[a] (95% CI)**								***P***
**Unadjusted**									
Definite PE (n = 4101, 4.93% PE)	1.17 (0.41, 3.33)	0.97 (0.31, 3.00)	1.76 (1.01, 3.10)	1.66 (0.94, 2.91)	1.21 (0.24, 6.07)	1.83 (0.55, 6.03)	1.72 (0.47, 6.29)	0.94 (0.43, 2.06)	0.14
**Adjusted[†]**									
Definite PE (n = 4101, 4.93% PE)	0.86 (0.27, 2.81)	0.93 (0.28, 3.06)	1.60 (0.91, 2.82)	1.65 (0.90, 3.05)	0.93 (0.16, 5.55)	1.73 (0.49, 6.06)	1.78 (0.45, 6.97)	1.03 (0.45, 2.36)	0.25

Note: PE, psychotic experiences; OR, odds ratio; 95% CI, 95% confidence interval; *P*, omnibus *P* value for association between psychotic experiences at age 12 years and cigarette/cannabis use classes.

[a] Compared to non-use class unless otherwise stated.

[†] Adjusted for sex, maternal education, emotional/behavioural problems (Strengths and Difficulties Questionnaire (SDQ) score age 9 years) and maternal cigarette smoking during pregnancy.

© 2017 Jones HJ et al. *JAMA Psychiatry.*

eTable 11. Associations between psychotic experiences at age 12 years and subsequent cigarette and/or cannabis use, limiting to non-users at age 12 years

Exposure	Early-onset cigarette	Early-onset cannabis	Late-onset cigarette	Late-onset cannabis	Early-onset cigarette versus Early-onset cannabis	Late-onset cigarette versus Early-onset cannabis	Late-onset cannabis versus Early-onset cannabis	Late-onset cannabis versus Late-onset cigarette	
	**OR[a] (95% CI)**								***P***
**All available data**									
Definite PE (n = 4700, 5.23% PE)	0.82 (0.27, 2.54)	1.16 (0.46, 2.93)	1.76 (1.05, 2.93)	1.69 (1.03, 2.79)	0.71 (0.15, 3.25)	1.51 (0.56, 4.07)	1.45 (0.50, 4.28)	0.96 (0.48, 1.94)	0.07
**Limiting to non-users at age 12 years**									
Definite PE (n = 4160, 5.00% PE)	0.17 (0.00, 48.24)	1.12 (0.26, 4.89)	1.46 (0.82, 2.60)	1.49 (0.86, 2.57)	0.15 (0.00, 61.07)	1.30 (0.28, 6.00)	1.32 (0.26, 6.64)	1.02 (0.46, 2.27)	0.42
**Limiting to non-users at age 12 years**									
**Unadjusted**									
Definite PE (n = 3646, 4.66% PE)	0.40 (0.02, 10.07)	0.28 (0.01, 14.89)	1.36 (0.72, 2.60)	1.46 (0.80, 2.68)	1.45 (0.01, 332.66)	4.91 (0.09, 267.58)	5.28 (0.09, 317.16)	1.07 (0.44, 2.63)	0.56
**Adjusted†**									
Definite PE (n = 3646, 4.66% PE)	0.20 (0.00, 24.59)	0.16 (0.00, 202)	1.23 (0.64, 2.39)	1.55 (0.82, 2.95)	1.19 (0.00, 8920)	7.53 (0.01, 8980)	9.48 (0.01, 13600)	1.26 (0.49, 3.23)	0.63

Note: PE, psychotic experiences; OR, odds ratio; 95% CI, 95% confidence interval; *P*, omnibus *P* value for association between psychotic experiences at age 12 years and cigarette/cannabis use classes.

[a] Compared to non-use class unless otherwise stated.

© 2017 Jones HJ et al. *JAMA Psychiatry.*

[†] Adjusted for sex, maternal education, emotional/behavioural problems (Strengths and Difficulties Questionnaire (SDQ) score age 9 years) and maternal cigarette smoking during pregnancy.

© 2017 Jones HJ et al. *JAMA Psychiatry*.

eFigure. Comparisons between 5-class models of cigarette/cannabis use patterns created using six and four time points

© 2017 Jones HJ et al. *JAMA Psychiatry*.

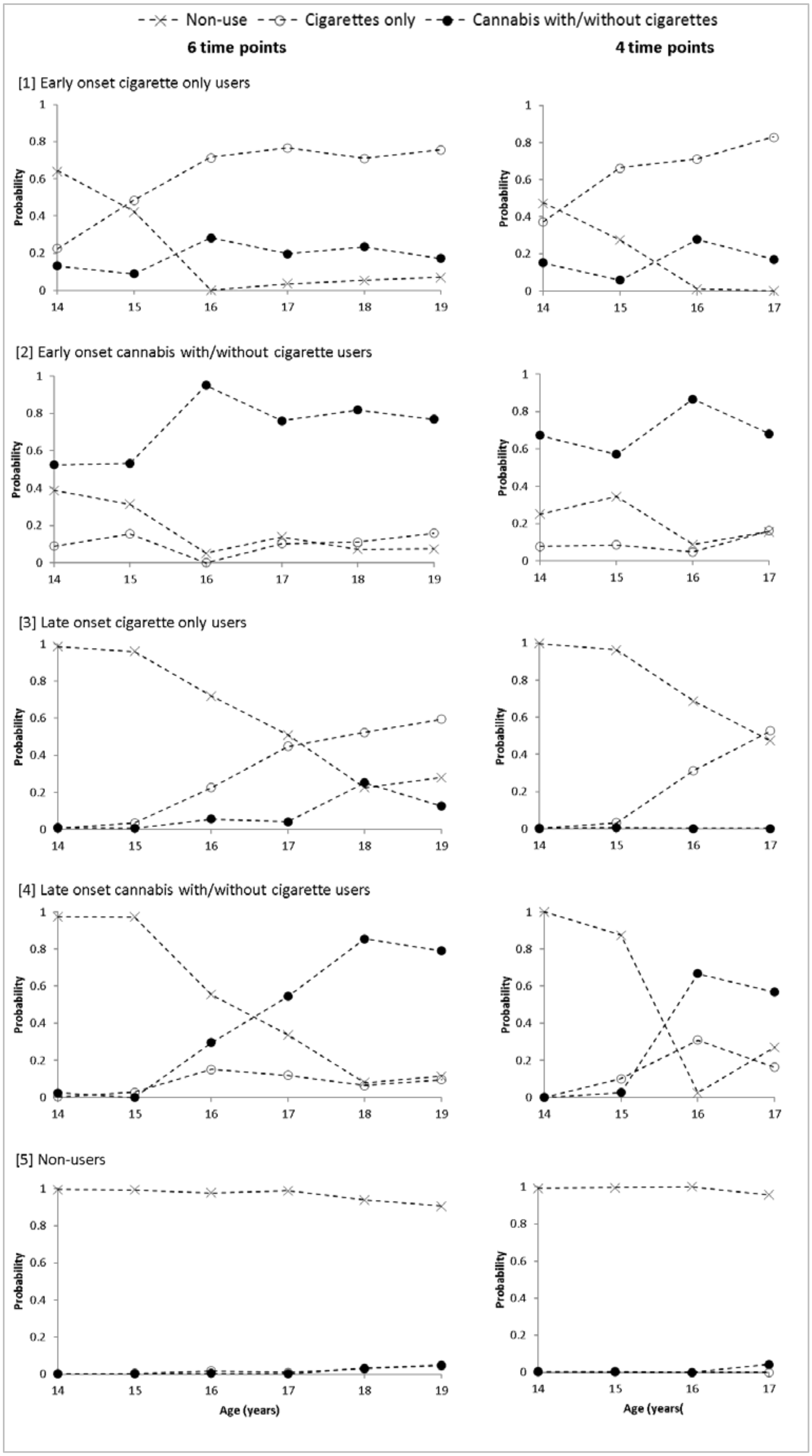

© 2017 Jones HJ et al. *JAMA Psychiatry.*

Note: The probability axis represents the probability of a class member being a non-user, a cigarette-only user or a cannabis with/without cigarette user at each time point.

© 2017 Jones HJ et al. *JAMA Psychiatry.*

eReferences

1. Horwood J, Salvi G, Thomas K, et al. IQ and non-clinical psychotic symptoms in 12-year-olds: results from the ALSPAC birth cohort. *British Journal of Psychiatry*. 2008;193:185-191.
2. Zammit S, Kounali D, Cannon M, et al. Psychotic experiences and psychotic disorders at age 18 in relation to psychotic experiences at age 12 in a longitudinal population-based cohort study. *Am J Psychiatry.* 2013;170:742-750.
3. Zammit S, Hamshere M, Dwyer S, et al. A population-based study of genetic variation and psychotic experiences in adolescents. *Schizophrenia Bull.* 2014;40(6):1254-1262.
4. Wechsler D, Golombok S, Rust J. *Wechsler Intelligence Scale for Children – Third Edition UK Manual.* Sidcup, UK: The Psychological Corporation; 1992.
5. Goodman R. The extended version of the strengths and difficulties questionnaire as a guide to child psychiatric caseness and consequent burden. *J Child Psychol Psyc.* 1999;40(5):791-799.
6. Crane C, Heron J, Gunnell D, Lewis G, Evans J, Williams JMG. Childhood traumatic events and adolescent overgeneral autobiographical memory: findings in a UK cohort. *J Behav Ther Exp Psy.* 2014;45(3):330-338.
7. Heron JE, Croudace TJ, Barker ED, Tilling K. A comparison of approaches for assessing covariate effects in latent class analysis. *Longitudinal and Life Course Studies.* 2015;6(4):15.
8. Lo Y, Mendell NR, Rubin DB. Testing the number of components in a normal mixture. *Biometrika.* 2001;88(3):767-778.

© 2017 Jones HJ et al. *JAMA Psychiatry.*
